# An Assessment of the Movement and Function of Children with Specific Learning Disabilities: A Review of Five Standardised Assessment Tools

**DOI:** 10.21315/mjms2020.27.2.3

**Published:** 2020-04-30

**Authors:** Nur Sakinah Baharudin, Dzalani Harun, Masne Kadar

**Affiliations:** Occupational Therapy Programme, Center for Rehabilitation and Special Needs Studies, Faculty of Health Sciences, Universiti Kebangsaan Malaysia, Kuala Lumpur, Malaysia

**Keywords:** assessment tool, children, function, movement, specific learning disabilities

## Abstract

Various standardised assessment tools have been used to evaluate children with disabilities. However, assessment tools that provide information on the movement and function of children with specific learning disabilities (SLD) are still limited. This article provides a narrative review of the characteristics of five movement and/or function assessment tools. The strengths and limitations of the tools will be highlighted. Empirical studies on the assessment tools used are reviewed based on three criteria: (i) standardised tools; (ii) assessment of movement and/or function; (iii) applicability to children with SLD ranging from 4–17 years of age and widely used in practice. The following instruments have been included as they have been found to fulfil the criteria: (i) the Bruininks-Oseretsky test of motor proficiency-2 (BOT-2); (ii) the movement assessment battery for children-2 (MABC-2); (iii) the pediatric balance scale (PBS); (iv) the Vineland adaptive behaviour scale-II (VABS-II) and (v) the pediatric evaluation of disability inventory-computerised adaptive test (PEDI-CAT). The article presents the characteristics, strengths and limitations of five standardised assessment tools that are currently in use, which measure the movement and/or function of children with SLD. This article concludes with a discussion of recommendations for the best approaches to evaluating the movement and functional abilities of children with SLD.

## Introduction

A specific learning disability (SLD) is a type of neurodevelopmental disorder. SLD is defined by the fifth edition of the *Diagnostic and Statistical Manual of Mental Disorders* (DSM-5) (1) as consisting of four key elements: i) characterised by having constant difficulty in learning and using one or more academic domains (i.e. reading comprehension, spelling, writing difficulties, mathematical reasoning) for at least six months although target skill interventions have been given; ii) the concerned academic skills are below what is expected at the individual’s age, which impairs functioning in school, at work and in activities of daily living (ADL); iii) SLD will be diagnosed at the age of onset during school-age or some people may appear the disorder when higher-level skills are demanded and iv) excluding those who have intellectual disabilities, visual or hearing impairments, mental disorders, neurological disorders, psychosocial-difficulties, language differences and who lack proficiency in the language of academic instruction (1).

Standardised assessment tools provide limited information on the movement and/or functional skill performance of children with SLD. Previous studies have mainly focused on assessing literacy problems in children such as reading (2) and other academic achievements (3–4). In addition, such studies have been developed and used by Western populations. The results of such studies have shown that movement and functional skill performance in school-aged children is lacking. Notably, the tasks item referring to function skills are less relevant to the cultural context in Malaysia. Therefore, it is necessary to select an appropriate movement and functions assessment for children with SLD in Malaysia.

The review focuses on evaluative measures to assess movement and functional performance in children with SLD. Aside from academic difficulties, children with SLD have often been found to exhibit clumsy and awkward movements while performing physical exercise (5). They show deficits in movement performance, such as in gross motor (6), fine motor (7), balance (8, 9) and functional skills (10). Insights into movement and functional performance of children with SLD are essential for health professionals and teachers to manage movement difficulty in these children. Therefore, the professionals involved are responsible for measuring motor proficiency in children with SLD (10–11).

Movement refers to the observable act of moving and demonstrates a change in the position of any part of the body (12). The performance of movement can be measured using motor skills to produce an output. Locomotors skills (e.g. running and hopping), manipulative or object control skills (e.g. catching and throwing) and balance skills (e.g. balancing and twisting) are fundamental movement skills required of school-aged children with SLD to deal with everyday life activities (13). Examples of movement tools used to assess children are the Bruininks-Oseretsky of motor proficiency test, second edition (BOT-2) (14) and the movement assessment battery for children, second edition (MABC-2) (15).

Function refers to performing an action or activities considered necessary for everyday life (16). For example, routine activities of daily living (ADL) include eating, grooming, toileting, dressing and functional mobility (16). There are several functional assessment tools available that measure ADL skills for children, such as the Vineland adaptive behaviour scale, second edition (VABS-II) (17) and the pediatric evaluation of disability inventory-computerised adaptive test (PEDI-CAT) (18).

This review explores some standardised tools for school-aged children with SLD in a clinical or educational setting. These tools are currently used widely in practice. Four of these tools are presently available in the *Occupational Therapy and Physical Therapy: A Resource and Planning Guide, 2nd edition* (19) for use by clinicians, occupational and physiotherapists, special education teachers, and/or parents. This review describes the characteristics of five tools and compares their strengths and limitations. The selected tools include: i) BOT-2; ii) MABC-2; iii) PBS; iii) VABS-II; iv) PEDI-CAT.

## Methods

Firstly, a comprehensive search of the literature has been conducted to identify existing paediatric tools. These tools are then evaluated to determine whether they meet the criteria for inclusion: i) standardised; ii) assess movement and/or function; iii) applicable to children with SLD ranging 4 to 17 years of age and iv) widely used in practice. Studies have been excluded if they are not currently available or if the review failed to find any literature on the tools. Finally, once the tools were included for review, complimentary publications were searched to enable a thorough evaluation of the tools. The selected studies describe the tools and their advantages or limitations in assessing children with SLD.

### Data Sources and Searches

**Figure 1:**
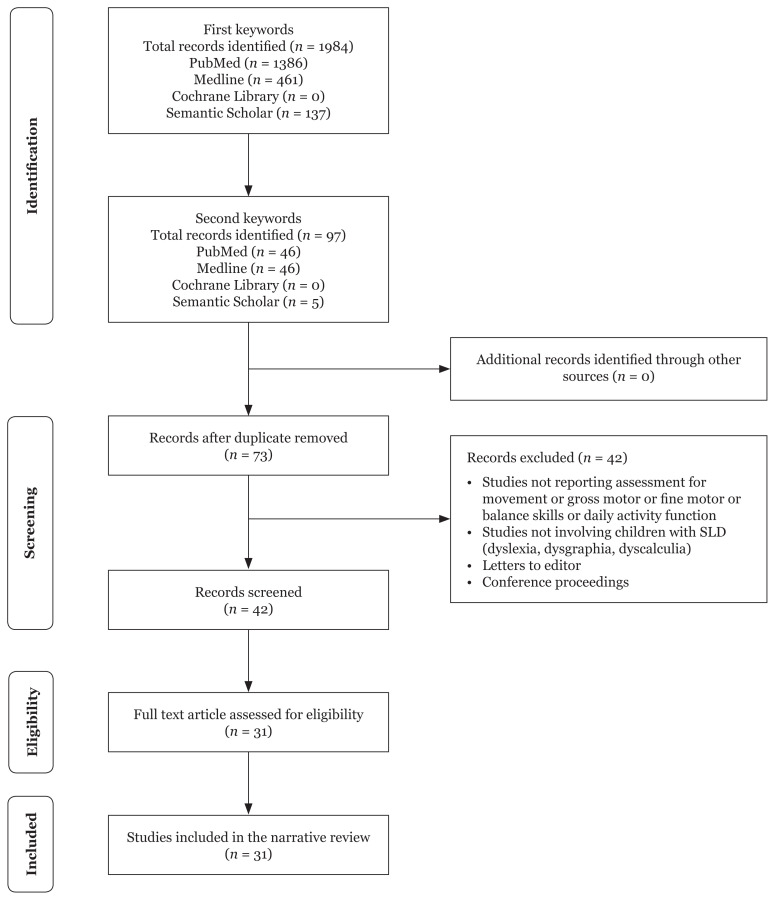
Flow chart of study selection

A literature review was conducted using the following databases: Medline, PubMed, EBSCOhost, OVID, ERIC and Google Scholar. The review looked at publications from 2000 to 2015. The first search used the following search term: Movement or gross motor or fine motor or balance and function or activity of daily living and assessment or evaluation or instrument and children or school-aged children and learning disabilities or learning disorders or SLD or dyslexia. The second search was performed to find supporting publications required for a further investigation of the selected tools. The search terms Identified assessment tool and learning disabilities or learning disorders or dyslexia have been used to search the available literature related to each tool. The flowchart of the article selections is shown in Figure 1.

## Results

### Characteristics of Movement and/or Function Assessment Tool

Five tools that meet the study’s inclusion criteria are: i) BOT-2; ii) MABC-2; iii) PBS; iv) VABS-II and v) PEDI-CAT. The administrative aspects of the reviewed tools such as the domains, age of children, duration of test administration, specific training required, administrator criteria, origin and cost are presented in Table 1.

## BOT-2

### Description

The BOT-2 (14), a revised version of the Bruininks-Oseretsky test of motor proficiency (BOTMP) (20), is used by education and health professionals to measure the performance of gross and fine motor skills in children aged 4–21 years. The test is proposed as both a screening tool and a diagnostic tool for children who may have motor impairments. It is also used for student selection in school placement and as an evaluative measure of the effectiveness of an intervention in movement and functional skills performance.

This tool consists of BOT-2 complete and short form. There are four motor area composites covered in the BOT-2 complete form including: i) fine manual control or FMC (subtests: fine motor precision and fine motor integration); ii) manual coordination or MC (subtests: manual dexterity and upper limb coordination); iii) body coordination (BC) (subtests: bilateral coordination and balance) and iv) strength and agility (SA) (subtests: running speed and agility and strength). However, the BOT-2 short form comprises 14 test items that are proportionately selected from the complete form.

The FMC refers to motor skill activities involving control and coordination of the distal musculature of the hands and fingers, such as cutting and copying shapes. The MC refers to motor skill activities involving control and coordination of the arms and hands, such as stringing blocks and dribbling a ball. The BC refers to control and coordination in posture and balance used by the large muscle groups such as jumping in place and standing one leg on a beam. Meanwhile, the SA covers aspects of fitness and coordination required in physical activity, such as running and sits-up.

Equipment and materials for administration the assessment tool, such as a manual, easel, record form and exam booklet, are provided with the purchase of a kit. The time required to administer the complete form varies from 45 min–60 min, whereas the short form takes about 15 min–20 min. An additional 10 min of preparation is required before conducting the assessment.

### Administration of the Test

The examiner is required to prepare the administration area before conducting the test. All required materials are placed accordingly and the child’s hand and foot preferences are determined. To begin the test, the examiner needs to follow all the administrative rules and teaching tasks. An optional administration book containing an image of a child performing the task may be placed in front of the person taking the test. The examiner uses verbal instructions and is allowed to demonstrate or provide support if necessary.

### Scoring Procedures

First, raw scores are obtained for each subtest, before each raw score is converted into a scale and a standard score. All information is obtained from the record book. Next, the sum of scores of four motor composites, i.e. FMC, MC, BC and SA, form a total motor composite. Total scores are then converted into an equivalent motor age and into descriptive categories (classification) of motor performance. Another section in the record book is the score profile. A confidence interval (90% and 95%) can be plotted by referring to the scale scores and standard scores. Otherwise, the pair-wise comparisons can be examined for statistical significance and frequency of difference.

## MABC-2

### Description

The MABC-2 (15), a revised version of the movement assessment battery for children (MABC) (21), is used to identify motor impairment and to provide a description of motor difficulties in children. It is also the test that is most frequently used by examiners to test the gross motor performance in children. The test is intended for use by both education and health professionals (22).

There are two forms of the MABC-2 that comprise the performance test and the checklist. The performance test is designed to assess fine and gross motor skill movement difficulties in children aged 3 to 16 years in three different age bands: i) Band 1: 3–6 years; ii) Band 2: 7–10 years and iii) Band 3: 11–16 years. Conversely, the checklist is used by parents, caregivers or teachers to rate how a child manages everyday tasks encountered at home and in school. Both forms are useful for identifying and describing motor function in children.

The MABC-2 contains three sections, which are manual dexterity (three items), ball skills-aiming and catching (two items), and static and dynamic balance (three items). The test kit consists of an intervention manual, checklists, record form, test materials and additional required equipment such as chairs, a table and a clipboard. Twenty to forty minutes to administer the performance test, while the checklist takes about 10 min to administer.

### Administration of the Test

To conduct the test, the examiner follows the task sequence according to the age bands in the examiner manual. However, the examiner may change the task order to maintain the interest and motivation of the children. The examiner is required to leave a note on the record form for future reference if the order is changed. Qualitative information on how the child approaches and performs the task is provided in the performance test. Trials for every task performance are given to the examinee before performing the task.

### Scoring Procedures

The first step in scoring the test items is to record the raw performance score. A score of ‘F’ is given if the child fails to complete the task, ‘I’ if the task is inappropriate, or ‘R’ if the child does not cooperate. Next, the raw performance score of each item is then converted into a standard score, with low standard scores indicating poor performance. However, according to the checklist high scores represent poor performance.

## PBS

### Description

The PBS (23), a paediatric version of Berg’s balance scale, is used to assess static and dynamic balance in school-age children aged 3–15 years with mild-to-moderate motor impairment. The test is intended for use by education and healthcare professionals for screening and evaluation.

This tool consists of 14 items pertaining to static and dynamic balance. The PBS requires equipment commonly found in schools and clinics, such as a height-adjustable bench, a chair with an armrest and back support, and a stopwatch. The test can be administered and scored in less than 20 min.

### Administration of the Test

To conduct the test, the examiner must demonstrate each task and explain the instructions that are given on the scoring sheet. The test allows for multiple trials of each item, and for the examinee to clarify the tasks both verbally and visually.

### Scoring Procedures

A score ranging from 0–4 is given for each item. The children are allowed for multiple trial on many of the items. The results should be rated according to the lowest standard that define the best performance of the child. For example, if during the first trial a child receives a maximum score of 4, then an additional trial need not be administered. The examiner may also choose to record the exact time in seconds for some scoring items. For items that pertain to balance, the subject is allowed to choose their preferred standing leg. Likewise, for items pertaining to reach, the subject is allowed to determined how far to stretch. A good balance performance is indicated by a higher score (24).

## VABS-II

### Description

The VABS-II (25) is a revision of the Vineland adaptive behaviour scale (Vineland ABS) (26). The tool is an individually administered instrument that measures a person’s adaptive level of functioning, which is used for both diagnostic and evaluative purposes. It is available in four forms, which are the survey interview form, the expanded interview form, the parent/caregiver rating form and the teacher rating form (TRF). This assessment is used from infancy to 90 years of age, except for TRF which is applicable to the age ranges of 3–21 years. The adaptive behaviour domain is conceptualised as encompassing the four broad dimensions of i) communication: receptive, expressive, written; ii) daily living skills: personal, domestic, community; iii) socialisation: interpersonal relationships, play and leisure time, coping skills; and iv) motor skills: fine motor and gross motor. In addition, the VABS-II also includes a maladaptive behaviour index: internalising and externalising, which the examiner may optionally assess.

The VABS-II takes approximately 20 min–60 min to be administered, depending on the adaptive levels exhibited by the person assessed. An additional 15 min–30 min are needed to hand-score the instrument.

### Administration of the Test

To conduct the test, the parents and/or caregivers are administered a semi-structured interview for the interview form (both survey and expanded). In contrast, both rating forms (parent/caregiver and teacher) need to be filled out independently using a provided rating scale.

### Scoring Procedures

A score of 0 indicates that the person never performs the skills independently, while 1 indicates that they ‘sometimes or partly perform skills independently’ and 2 indicates that they ‘often perform skills independently’. Individual items are rated. All sub domain item scores are totalled and raw scores are transformed into a scale score, domain score and adaptive behaviour composite.

## PEDI-CAT

### Description

The PEDI-CAT (18) is a revision of the pediatric evaluation of disability inventory (27) into a computerised-adaptive test (CAT) and is used to measure the functional skills of children and youth from infancy to 20 years of age. The PEDI-CAT is designed for use by health professionals, such as occupational therapists and physiotherapists, as well as professional educators in schools. The test is specifically designed for some clinical use, for example, a screening tool to detect functional delay, or as an evaluative measure and observation of individual change in a child. There are two types of PEDI-CAT, which include speedy (precision) CAT and content-balanced (comprehensive) CAT. The speedy CAT gives the fastest results as it consists of 5–15 items per domain, whereas the content-balanced CAT consists of approximately 30 items per domain. There are a total of 271 items in four domains, including daily activities, mobility, social/cognitive and responsibilities. The PEDI-CAT requires equipment such as a computer or iPad installed with CAT software, a table, and a chair. It takes approximately 15 min–20 min to complete the items in the content-balanced CAT assessment.

### Administration of the Test

Parents/caregivers can complete the PEDI-CAT independently through a structured interview or by professional judgment. To begin the test using the Windows version, the interviewer must first enter the identification number of the child and select the domain that is being assessed. Then, the interviewer selects the required demographic information such as gender, date of birth and types of mobility. Next, the interviewee must respond to the items that appear on the screen. About 20 min are required to complete all questions.

### Scoring Procedures

The test uses a 4-point difficulty scale with a response of 1 indicating ‘unable’, 2 indicating ‘hard’, 3 indicating ‘a little hard’ and 4 indicating ‘easy’. The original PEDI is scored using a two-point response: ‘unable or capable’. The PEDI-CAT provides two types of summary scores calculated for each domain. A normative score is provided as age percentile and T score. These results are based on a child’s chronological age and the child’s functioning is interpreted in relation to others of the same age. A scaled score provides the current functional skills of a child and progress over time. Additionally, an item map is presented if the subject is using a content-balanced CAT. The map will represent a sequential pattern of functional skills consistent with a child development.

## A Review of the Strengths of the Movement and/or Function Assessment Tools

The strengths of the tools include their reliability and validity, cross-cultural applications, standardisation, and special features/characteristics. A summary of the strengths is presented in Table 2.

### Reliability and Validity

Generally, evidence shows that a lack of information exists on the reliability and validity of the selected tools for children with SLD. However, the review of the five tools, i.e. BOT-2, MABC-2, PBS, VABS-II and PEDI-CAT demonstrates that the tools have some strong psychometric properties.

Findings from a reliability study show that the BOT-2 is reported to have moderate to strong inter-rater and test-retest reliability for both complete and short forms (28). The BOT-2 shows split-half reliability for internal consistency and reliability coefficients for the subscale, composite, total motor composite and short form scores that range from high 0.70 s to mid-0.90 s (28).

For MABC-2, in the two oldest age bands, the inter-rater and test-retest reliability shows an intra-class correlation (ICC) range from *r* = 0.92–1.00 and between *r* = 0.62 and 0.92, except for one item (29).

Similarly, the PBS has good reliability in assessing balance with excellent test-retest reliability, i.e. the ICC coefficient is 0.82–0.93, inter-rater reliability, ICC is 0.96–0.99 and internal consistency, Cronbach’s α is 0. 89–0.97 (23).

The test-retest reliability of VABS-II has been found to be high: *r* = 0.95–0.99 for all domains and adaptive behaviour composite showing ICC = 0.99; *r* = 0.74 (*n* = 160, 6–18 years, standardisation sample) is reported in Sparrow et al. (26).

For PEDI-CAT, previous studies have shown that test-retest reliability for all four domains is high (ICC = 0.96–0.99) (30). However, the reliability of the reviewed assessment tools is well developed compared to its validity.

Content and concurrent validity is the common validity that has been established. For example, the BOT-2 has established content validity (31). For MABC-2, the concurrent validity with the BOTMP *r* = −0.53 and with Korperkoordinationtest fur Kinder (KTK) *r* = 0.62 (32).

### Cross Cultural Applicability

The Dutch translation of the MABC-2 checklist shows a construct validity with a Cronbach’s alpha score of 0.94 (33). The total score of the Brazilian-Portuguese PBS version also shows excellent intra-rater reliability with an ICC, 0.85 and inter-rater reliability with an ICC, 0.91 (24). In addition, the survey form of VABS-II was translated into a Hindi version and has strong validity and reliability (34). A Spanish version of the survey interview form and parent/caregiver rating form is also available (35). PEDI has been translated into multiple languages, including Dutch, Norwegian, Swedish, Spanish (United-States), Portuguese (Brazil), Slovene, Turkish, Icelandic, French (Canada), Hebrew, Japanese and Chinese (17). PEDI-CAT is available in Spanish.

### Standardisation

Most of the assessments in the review have normative norms, which are represented by the United States population (BOT-2, VABS, PEDI-CAT) and European children (MABC-2). The PBS refers to criterion norms (34). The standardisation helps to determine movement and/or functional performance of a child individually and in relation to the general population at a similar age.

### Special Features or Characteristics

There are a few special features or characteristics of the tools that can be observed in this review.

#### Age specificity

All tools were appropriate for school-aged children ranging from 4–17 years of age.

#### The use of photos in the test

The BOT-2 is a standardised tool that provides photos in each subtest to allow for a standard and effective administration of the test (28). This tool has proven to be transparent and relevant to childhood motor activities such as ball skills, movement, paper/pencil tasks, and card sorting (28).

#### The user-friendly assessment tool

The MABC-2 is designed to be user friendly, is easy to administer, and is very applicable to educational settings (36). It is recommended that the MABC-2 be used as a screening tool for motor impairment due to its simple test administration (37–38). Moreover, the MABC-2 requires minimal training and is commonly used due to its sound psychometric properties (39). Furthermore, a short administration time (20 min) is an advantage for children with a short attention span, and therefore, this test is widely used to measure motor performance (40). The game-like motor tasks for BOT-2 are able to capture the child’s interest and the verbal instructions are easily understood (31). Therefore, the BOT-2 is suitable for children who do not speak English as a native language. The complete version of BOT-2 has a total of 53 items, compared to 46 items in BOTMP (39) Therefore, a wider range of motor tasks can be evaluated using this tool, including gross motor, fine motor and balance skills.

#### Less costly assessment tool

The PBS is one of the standardised protocols for testing the balance of children with disabilities. The tool is able to distinguish between children who are developing normally and children with mild motor impairments (41). This test is inexpensive, can be downloaded at no cost, and the equipment needed to administer the test is easily available in schools, clinics, or hospital settings (42).

#### Broad sensitivity for ages and abilities

The VABS-II provides an assessment of adaptive functioning across a broad range of ages, i.e. from 0–90 years old and is suitable for school-aged children (24). The VABS-II provides different forms for teachers and parents to report any developmental problems that children may have (35). This tool also has a broad sensitivity across ages and ability levels. Age-equivalent scores are used, which provides an advantage when comparing performance domains.

#### Computerised-scoring programme

The VABS-II consists of a computerised-scoring programme that makes the computation and interpretation of scores much easier and may avoid problems that can arise in manual scoring. The scoring instructions are nicely formatted and easy to follow and the manual contains several examples of completed protocols with annotations of how to discontinue standards, how to calculate raw scores on each scale and how standardised scores are obtained based on raw scale scores. The PEDI-CAT is self-contained, and can be used to assess separate domains such as daily activities, mobility, social/cognitive and responsibilities (43). The PEDI-CAT is designed with clear and simple instructions, and illustrations are provided for each item to improve clarity (43). Finally, the new 4-point rating scale: ‘Unable’, ‘Hard’, ‘A little hard’ and ‘Easy’, has increased the precision of the PEDI-CAT rather than ‘capable/not capable’ in the old version of PEDI (43).

## A Review of the Limitations of the Movement and/or Function Assessment Tools

The limitations highlight the examiner, the administration of the test, and the scoring procedures. The summary of the limitations for the five reviewed tools are presented in Table 3.

### Examiner

The biggest limitation pertaining to the examiners is that they are required to have high qualifications or training to use some tools, especially to score and interpret the results. Some also report that a tool such as the BOT-2 is hard to obtain (31, 44).

### Administration of the Test

In using BOT-2 to test a shuttle run, an open/large room is needed in order to complete the 50-foot shuttle run in under 13 s and this can be a challenge (31, 44). Moreover, the time required to complete one comprehensive test in BOT-2 is rather long (45 min–60 min). This may pose a challenge to children below three years of age who are participating in the assessment (39, 44).

MABC-2 requires that only the equipment supplied in the test kit be used. Any change may invalidate the results obtained (40). In addition, the tool does not cover the full range of motor abilities in children due to the fact that there are only eight tasks in each domain being assessed (12). Furthermore, the time duration of administration (20 min–30 min for eight items) is an unacceptable proportion and is too long (45). This test is also limited to certain age bands and age skills (45). Finally, repeated trials (5 practice and 10 test trials) can be a challenge for some children who may become too tired to perform the tasks. A longitudinal analysis of MABC-2 is difficult to conduct due to different tasks and age bands (46). Furthermore, there are no separate norms for boys and girls in MABC-2 (46).

The PBS is influenced by height. Examples of this barrier are found in the items sit to stand, transfers, turning to look behind, retrieving an object from the floor and reaching forward (42). During the transfer technique, most children with a shorter statue will wiggle back into their chair. Therefore, it is not possible for these children to achieve a full score for this item. The PBS also tests static balance but not locomotive balance or overhead reaching (42). Therefore, dynamic balance cannot be tested using this tool.

The VABS report does not provide a self-report form and is lengthier than some other measures of adaptive functioning (24).

The reported assessment limitations using PEDI-CAT include items in the responsibility domain that require children to use a combination of functions to carry out life tasks irrelevant of their age. They also include tasks that do not allow a particular activity to be completed, and certain tasks that are simply too difficult for younger children to complete. Furthermore, certain activities simply do not apply to a particular child and family, and are not applicable to older children (30). For this reason, parents may find this domain and it’s given questions difficult to respond to. Moreover, some parents may require assistance in using the computer (30). There is also a lack of information on the cross-cultural differences that exist around using the tools in children with SLD. Many studies have reported challenges conducting PEDI due to issues of cross-cultural applicability i.e. problems with language translation, cultural differences in some activities, and different beliefs pertaining to encouraging a child to perform certain activities (17). Additionally, one of the largest difficulties regarding translation is finding suitable words in different languages. For example, Berg et al. (47) report difficulty in translating ‘prompting’, ‘fasteners’ and ‘item’ into Norwegian words. In addition, the use of a bathtub is not common in some countries, as reported by Norwegian and Dutch teams (17). Lastly, different parenting experiences in normative data between America and other countries produce different results (47).

### Scoring Procedures

Previous studies report that the scoring conversion system in BOT-2 is quite complicated and may provide a challenge to the examiner during the scoring process (28, 39). This is particularly true with regard to confusion that arises when using the BOT-2 record form and test manual (28).

## Discussion

To date, there are many assessment tools available to assess the movement and/or function of children with learning disabilities (14–15, 17–18). Overall, each assessment tool in the articles reviewed was used to measure the movement or functional skills performance of school-aged children with SLD. Only the PBS is freely available online, while other assessments must be bought at a fairly expensive price with an authorised dealer and need training to administer. All recent assessment tools also were developed in the English language. Therefore, thorough translation work and validation studies must be conducted before such tools are used in different cultural contexts (48). Several first spoken languages exist in Malaysia including Bahasa Melayu, Chinese, Tamil, Kadazan-Dusun and Jaku-Iban (49). Therefore, a functions assessment tool, which usually requires parents to use a rating scale, may pose a challenge to some parents who may not be able to understand or interpret the item tasks.

These five assessment tools were standardised and have strong reliability and validity. Some of the assessment tools were translated to other ethnic-language version such as Norwegian (Dutch), Brazilian (Portuguese), Hindi and Spanish. Certain unique features were designed for each tool, such as graphics, game-like motor tasks and user-friendly aspects, to ensure that the tools were useable for children with learning disabilities.

Unfortunately, some limitations exist regarding the administration of certain tools, which may disadvantage some people if they used to assess the movement and functional skills performance of children with SLD. For example, the BOT-2 is very difficult to obtain and a large space is needed to conduct the assessment. Item tasks in the MABC-2 and VABS-II also require a lot of time and repeated trials, which were not appropriate especially for young children. This review recommends that assessment tools with a shorter administration time are used with young children, such as pre-school aged children. A long test duration may pose challenges to younger children in participating throughout the duration of the assessment (45), and the test may have to be administered over multiple sessions due to a child’s fatigue. Some item tasks in the PEDI-CAT were irrelevant according to age, not culturally relevant, or the child do not have the opportunity to participate to the activity yet. Therefore, this may pose challenges to parents who are rating the scores of these tests.

Many different factors influence the selection of assessments of children with SLD. According to Cools et al. (45), certain criteria for the selection of assessments of children should be considered, such as the purpose of assessment. For example, the evaluative measure of general motor proficiency, fine or gross motor proficiency assessment, and the prevalence of assessment must be specified. Secondly, the test must be appropriate and specific to the target population. Moreover, the test must be easy to understand and administer. Lastly, differences exist between the assessments regarding cultural norms and cultural similarities. Therefore, this review recommends using a suitable assessment tool which is standardised, relevant and specific to the target population, and one that can be understood by the raters and acceptable to the specific population norms.

## Conclusion

In conclusion, the primary goal of the review is to present the characteristics, strengths and limitations of five recent standardised assessment tools in practice, which measure the movements and/or functional skills of children with SLD. A summary of the characteristics, strengths and limitations of these assessment tools can be found in Table 3.

## Figures and Tables

**Table 1:** Administrative aspects of movement and/or function assessment tools

Movement and/or function assessment	Movement, function or both	Domain	Age of children (years)	Duration of test (min)	Specific training required	Administrator criteria	Origin	Cost of the tools (USD)
**BOT-2 (14)**	Movement	Fine manual control, manual coordination, body coordination & strength and agility	4–21	Complete form: 45–60; Short form: 15–20	Yes	Health professionals/special educators	USA	1,975.00
**MABC-2 (15)**	Movement	Manual dexterity, Ball skills-aiming and catching, & static and dynamic balance	3–16	20–30	Yes	Health professionals/special educators	USA	1,859.00
**PBS (23)**	Movement	Static balance & dynamic balance	3–15	< 20	No	Health professionals/special educators	USA	Freely available online
**VABS-II (25)**	Function	Communication, daily living skills, socialisation, motor skills & maladaptive behaviour index (optional domain)	Birth–90	Interview format: (20–60) Self-report: (30–60)	Yes	Health professionals/special educators	USA	Expended set: 203.00 Survey form & expended set: 360.00 Teacher complete rating form: 431.00
**PEDI-CAT (18)**	Both	Daily activity, mobility, social/cognitive & responsibility (optional domain)	4–21	< 20	Yes	Health professionals/special educators/parents	USA	418.00

**Table 2:** The strength of the movement and/or assessment tools

Tools	Reliability & Validity	Cross-culturality	Standardisation	Special features/characteristics
**BOT-2 (14)**	Strong inter-rater and test-retest reliability Internal consistency is high ranging from 0.70 to 0.90 Has established content validity	Available for Norwegian (Dutch) version for MABC-2 checklist	Normative norms	Age appropriate for school-aged children Use of photos to enable a standard administration It involves game-like motor tasks and instructions were not complicated Suitable for children of non-English speaking backgrounds Age and specific norms for individuals from 4 to 21 years of agr A total of 53 motor tasks
**MABC-2 (15)**	Two oldest age bands showing inter-rater from 0.92 to 1.00 and test-retest from 0.62 to 0.92 Concurrent validity MABC-2 with the BOTMP is -0.53, and KTK is 0.62		Normative norms	Age appropriate for school-aged children Easy to use and very usable in educational settings Used as a screening tool for motor impairment Requires minimal training to administer Easier to administer to children with short attention spans
**PBS (23)**	Test-retest reliability from 0.82 to 0.93 Inter-rater reliability is 0.96 to 0.99 Internal consistency is 0.89 to 0.97	Available in a Brazilian-Portuguese version	Criterion norms	Age appropriate for school-aged children A standardised protocol for balance testing More appropriate for a child’s developing balance skills Can use equipment commonly found in schools and clinics Inexpensive
**VABS-II (25)**	Test-test reliability is 0.95 to 0.99 for domain Test-retest reliability is 0.99 for adaptive behaviour composite	The Survey form is available in Hindi and Spanish versions The parent/caregiver rating form is available in a Spanish version	Normative norms	Age appropriate for school-aged children Provides an assessment for adaptive functioning across a broad range of ages and abilities Has different types of forms: Survey interview form, expanded interview form, survey (parent/caregiver) rating form, teacher rating form Use of age equivalent scores, rather than standard scores Has a computerised scoring programme
**PEDI-CAT (18)**	Test-retest is 0.96 to 0.99 for all four domains	CAT is available in Spanish	Normative norms	Age appropriate for school-aged children Reducing test burden by minimising irrelevant test items administered (from 30 items per domain to 15 items per domain) Self-contained and can be use separately Items are worded using everyday language and clear examples Illustration of daily activities and mobility items are included The new four-point rating scale: ‘Unable’, ‘Hard’, ‘A Little Hard’ and ‘Easy’ has increase the precision of the PEDI-CAT Has a computerised scoring programme

**Table 3:** The limitations of the movement and/or assessment tools

Tools	Examiner/Evaluator	Administration of the test	Scoring procedure
**BOT-2 (14)**	Require a high qualification and training for the examiner.	Difficult to obtain the assessment tool. A large test room/open space of 50-foot/15 m is required. Longer duration of administration between 45 min and 60 min (complete form).	The scoring conversion system is quite complicated
**MABC-2 (15)**		Only the equipment supplied in the test kit must be used. Any change may invalidate the results obtained Does not cover the full range of motor abilities that might be implicated Time duration is too long for eight items Limited to a certain age band The repeated trials (5 practice and 10 test trials) can be a challenge for some children	
**PBS (23)**		Most of the test items are influenced by height: sit to stand, transfers, turning to look behind, retrieving the object from the floor, and reaching forward Does not test locomotive balance Does not test overhead reaching	
**VABS-II (25)**		Does not provide a self-report form Lengthier than some other measures of adaptive functioning	
**PEDI-CAT (18)**		The items in the responsibility domain require children to use several functional skills in combination to carry out life tasks Some items were irrelevant according to the child’s age Parents are not exposed for a particular activity for their children The activities simply did not apply to their child and family Some activities are not applicable to children over a certain age Assistance is required for some parents to use the computer Challenges conducting PEDI due to cross-cultural applicability	
